# Supplementary material for: CIC-DUX4 Chromatin Profiling Reveals New Epigenetic Dependencies and Actionable Therapeutic Targets in CIC-Rearranged Sarcomas
Source: Cancers (Basel). 2024 Jan 21;16(2):457. doi: 10.3390/cancers16020457 (PMC10814785; doi:10.3390/cancers16020457)
Supplement: Supplementary file 1 [file cancers-16-00457-s001.zip › cancers-2782420-supplementary.pdf]

Supplementary Figures 1-3

Figure S1

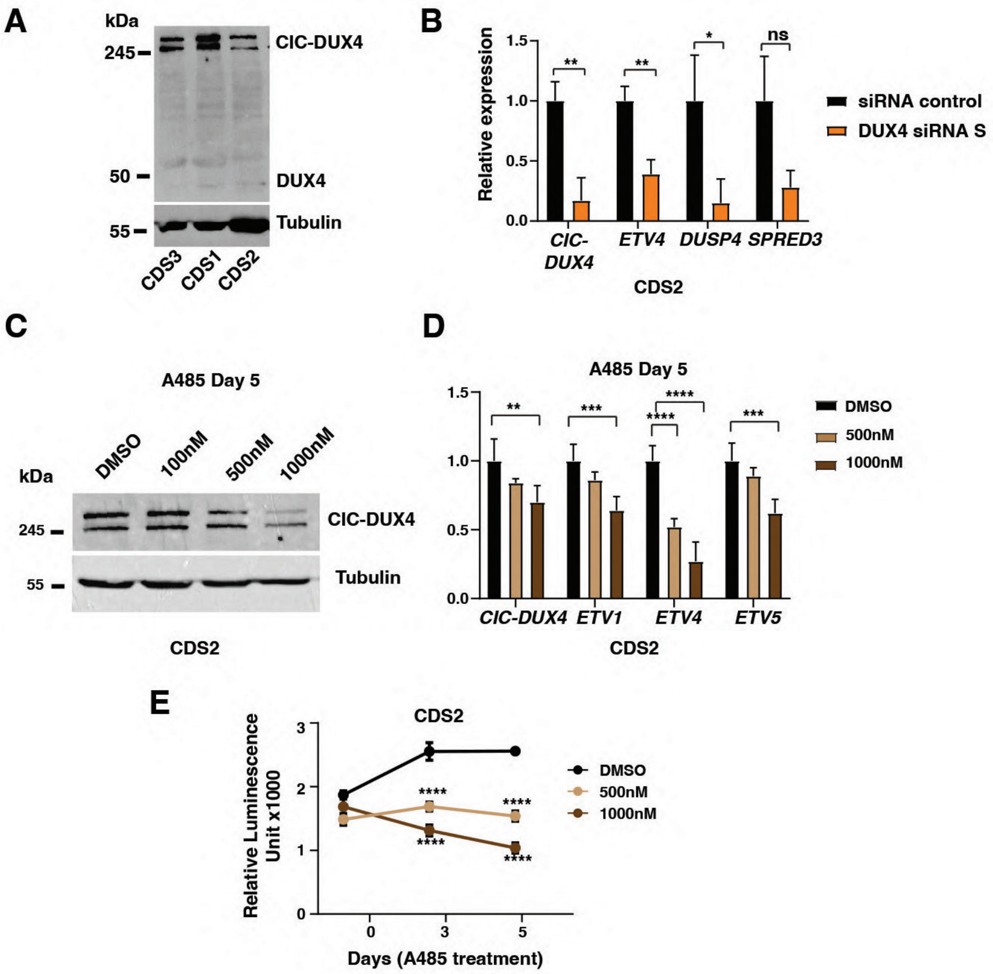

Figure S1: (A) Western Blot showing CIC-DUX4 as well as endogenous DUX4 protein levels across CDS1, CDS2 and CDS3 cell lines. **(B)** RT-qPCR analysis of the expression levels for a panel of established CIC-DUX4 target genes upon the depletion of the fusion protein in CDS2 cells. Data are presented as mean +/- SD, with n=3 per group. Gene expression levels were compared by two-way ANOVA. **(C)** Western Blot showing the decrease in CIC-DUX4 protein levels in CDS2 cells treated with increasing concentrations of A-485. **(D)** RT-qPCR analysis of the expression levels for CIC-DUX4 and a panel of fusion protein target genes in the CDS2 cell line treated with two different concentrations of A-485. Data are presented as mean +/- SD, with n=3 per group. Gene expression levels were compared by two-way ANOVA. **(E)** Changes in CDS1 proliferation upon treatment with increasing concentration of A-485, as assessed by luminescence. Data are presented as mean +/- SD, with n=4 per group. Statistical analyses were done using two-way ANOVA.

Figure S2

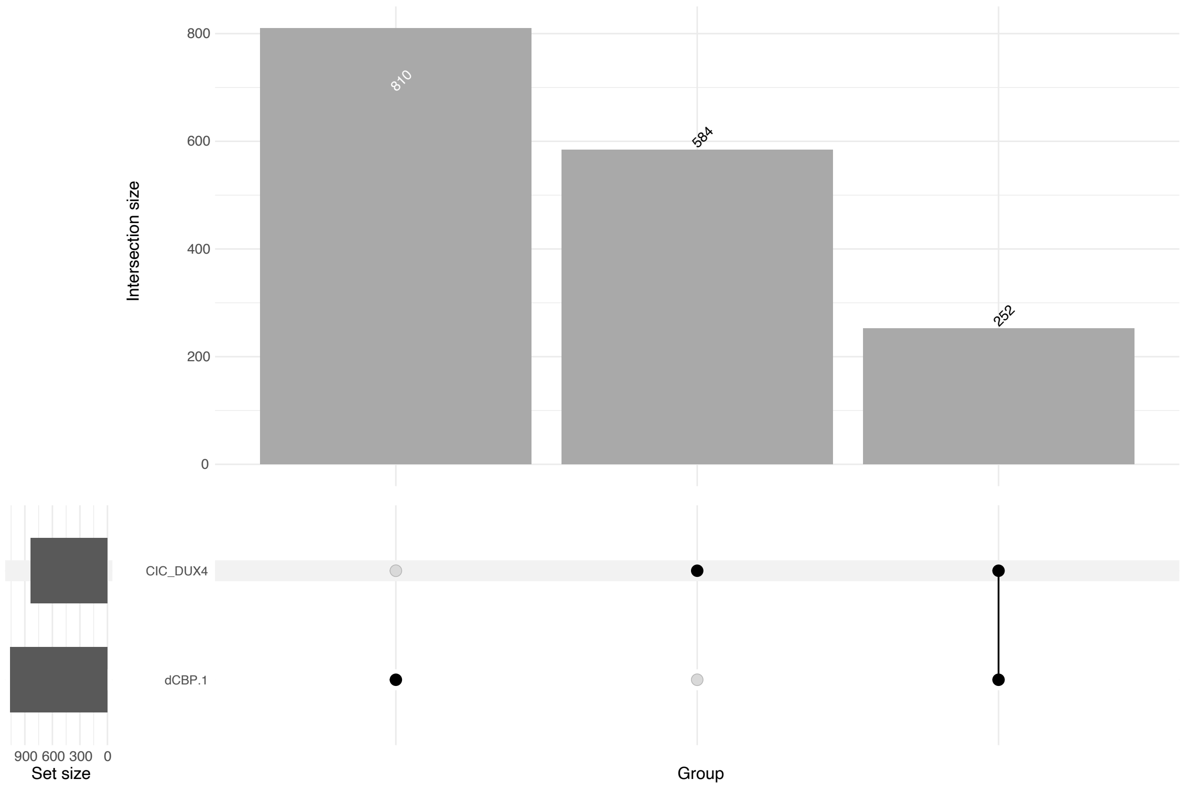

Figure S2 :Upset plot showing the number of common downregulated genes in CDS2 following CIC-DUX4 depletion or dCBP-1 treatment and their intersection. Vertical bars show the number common genes (intersection size) from a given set of methods (bottom filled connected circles). Horizontal bars illustrate the total number of genes (set size) output from each integration method.

Figure S3

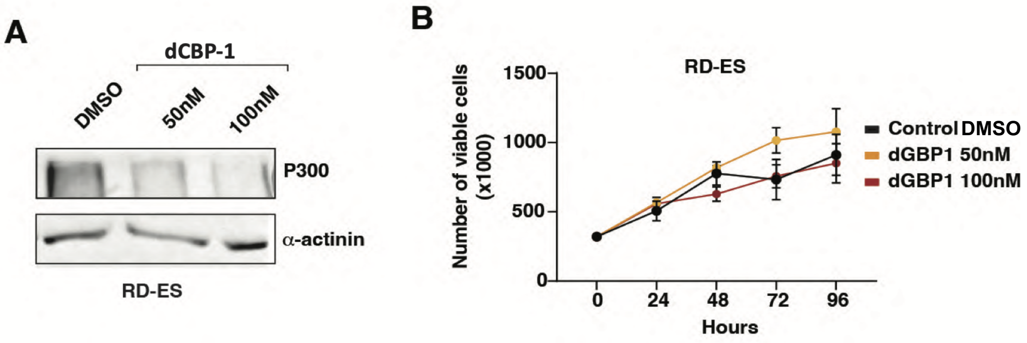

Figure S3: **(A)** Western blot showing the decrease in p300 protein levels in the RD-ES Ewing sarcoma cell line treated with increasing concentrations of the p300 protein degrader dCBP-1. **(B)** Plots depicting the lack of changes in cellular proliferation upon treatment of the RD-ES line with two different concentrations of dCBP-1, as assessed by relative luminescence. Data are presented as mean +/- SD, with n=4 per group. Statistical analyses were done using two-way ANOVA.

Supplementary Tables 1-6

Table S1. PCR primers

Gene	Forward primer	Reverse primer
GAPDH	5′-GTCTCTCTGACTTCAACAGCG-3′	5′-ACCACCCTGTTGCTGTAGCCAA-3′
TBP	5′-CGGCTGTTTAACTTCGCTTC-3′	5′-CACACGCCAAGAAACAGTGA-3′
CIC-DUX4(CDS1)	5′-CTCACCCAGCTCGGACTCT-3′	5′-ACTCCCCTGGGACGTGG-3′
CIC-DUX4 (CDS2)	5′-CTCACCCAGCTCGGACTCT-3′	5′-GGAGTGCAGACCAGG-3′
EP300	5′-GCTTCAGACAAGTCTTGGCAT-3′	5′-ACTACCAGATCGCAGCAATTC-3′
ETV1	5′-GCAAGAACGCTTCCTGGC-3′	5′-CCTTCCCGATACATTCCT-3'
ETV4	5′-AGGAACAGACGGACTTCGCCTA-3′	5′-CTGGGAATGGTCGCAGAGGTT-3′
ETV5	5′-TCAGCAAGTCCCTTTTATGGTC-3′	5′-GCTCTTCAGAATCGTGAGCCA-3′

Table S2. Primary antibodies for Western blot

Antibodies	Source	Identifier
Rabbit polyclonal anti-DUX4 C-terminal	Origene	AP51343PU-N
Rabbit monoclonal p300 (D2X6N)	Cell signaling	54062
Mouse monoclonal anti-α-actinin	Sigma-aldrich	05-384
Mouse monoclonal anti-α-tubulin	Sigma-aldrich	T6074-100UL

Table S3. Antibodies for Chromatin immunoprecipitation

Antibodies	Source	Identifier
Rabbit polyclonal anti-DUX4 C-terminal	Origene	AP51343PU-N
Rabbit monoclonal p300 (D2X6N)	Cell signaling	54062
Anti-trimethyl-Histone H3 (Lys4)	Merck	07-473
Histone H3K27ac	Active Motif	39133
Anti-Histone H3 (mono methyl K4), ChIP grade	Abcam	ab8895

Table S4. CIC-DUX4 peaks

Chromosome number	Genome coordinates		Relation to neighboring genes
**chr1**	12689039	12689881	Distal
**chr1**	41921326	41922608	Distal
**chr1**	42420190	42422423	Distal
**chr1**	43146903	43147846	TSS
**chr1**	46268094	46269083	TSS
**chr1**	51435413	51438760	TSS
**chr1**	52522370	52524108	TSS
**chr1**	53704332	53707143	TSS
**chr1**	58870890	58872591	Distal
**chr1**	58906674	58908865	Distal
**chr1**	65935448	65938537	Distal
**chr1**	65992044	65994045	TSS
**chr1**	78443353	78445167	TSS
**chr1**	78469209	78470440	TSS
**chr1**	86045185	86046336	TSS
**chr1**	89196766	89197894	Distal
**chr1**	92149449	92150981	Distal
**chr1**	92248675	92250890	Distal
**chr1**	94041220	94042981	Distal
**chr1**	120557180	120558858	TSS
**chr1**	150207989	150209405	TSS
**chr1**	150335241	150337010	TSS
**chr1**	150337231	150339148	TSS
**chr1**	150539465	150541648	TSS
**chr1**	151042700	151043421	TSS
**chr1**	151952315	151954319	Distal
**chr1**	153853077	153854927	TSS
**chr1**	153895592	153896682	TSS
**chr1**	156570542	156571619	TSS
**chr1**	160255021	160255268	TSS
**chr1**	161015509	161016473	TSS
**chr1**	161102644	161103702	TSS
**chr1**	168434999	168437326	Distal

**chr1**	168551210	168552768	Distal
**chr1**	172428544	172431474	Distal
**chr1**	181068266	181069409	TSS
**chr1**	183805782	183806939	Distal
**chr1**	184579985	184580965	TSS
**chr1**	204475496	204477414	TSS
**chr1**	205719317	205720379	TSS
**chr1**	209757074	209759035	TSS
**chr1**	209759272	209760635	TSS
**chr1**	209770647	209771591	Distal
**chr1**	209788716	209790725	Distal
**chr1**	209791597	209794239	Distal
**chr1**	234736124	234744261	TSS
**chr1**	234859134	234860739	TSS
**chr1**	235461488	235462805	Distal
**chr1**	244999185	245000121	TSS
**chr1**	249239829	249240600	Distal
**chr2**	1569954	1571366	Distal
**chr2**	1628916	1630464	Distal
**chr2**	1655699	1662171	Distal
**chr2**	1681525	1685252	Distal
**chr2**	1701717	1705128	Distal
**chr2**	1705566	1706572	Distal
**chr2**	1709718	1712864	TSS
**chr2**	1718624	1719977	TSS
**chr2**	1720376	1722561	TSS
**chr2**	1724008	1729975	TSS
**chr2**	1730308	1731930	TSS
**chr2**	1732590	1737096	TSS
**chr2**	1740737	1742194	TSS
**chr2**	1742509	1743697	TSS
**chr2**	1745278	1747082	TSS
**chr2**	1748936	1754243	TSS
**chr2**	1754499	1757667	Distal
**chr2**	1758604	1760444	Distal
**chr2**	3644049	3645445	TSS
**chr2**	9548807	9549683	Distal
**chr2**	12316345	12318460	Distal
**chr2**	20101425	20102656	TSS
**chr2**	24296284	24299552	TSS
**chr2**	25351897	25352165	Distal
**chr2**	27273417	27274369	TSS
**chr2**	27545585	27546807	TSS
**chr2**	27849149	27850533	TSS
**chr2**	31541726	31543125	Distal
**chr2**	38385237	38386267	Distal
**chr2**	43985761	43987438	Distal
**chr2**	46561905	46563505	TSS
**chr2**	47417896	47421277	TSS
**chr2**	47496272	47498958	Distal
**chr2**	48094241	48095339	Distal
**chr2**	65527835	65530319	Distal
**chr2**	65560535	65561094	Distal
**chr2**	65576481	65579175	Distal
**chr2**	65640458	65641446	Distal
**chr2**	65655365	65657706	TSS
**chr2**	65661145	65662872	TSS
**chr2**	65663490	65665550	TSS
**chr2**	67663655	67665016	Distal
**chr2**	88927387	88928046	TSS
**chr2**	98485316	98486465	Distal
**chr2**	98750201	98751716	Distal
**chr2**	109294530	109295353	Distal
**chr2**	134474050	134477233	Distal
**chr2**	149403463	149404168	TSS
**chr2**	179143396	179144604	Distal
**chr2**	197789536	197790884	TSS
**chr2**	201753453	201754963	TSS
**chr2**	203130105	203132728	TSS
**chr2**	207997786	208001149	TSS
**chr2**	208575219	208576068	TSS
**chr2**	216979701	216981012	TSS
**chr2**	224588362	224590900	TSS
**chr2**	238623947	238625292	Distal
**chr3**	9439954	9442051	TSS
**chr3**	9807973	9811293	TSS
**chr3**	53914692	53915668	TSS
**chr3**	57541167	57542040	TSS
**chr3**	63953431	63954652	TSS

**chr3**	78880626	78882281	Distal
**chr3**	99268061	99270931	Distal
**chr3**	99427444	99440050	Distal
**chr3**	99535602	99536948	TSS
**chr3**	101292627	101292865	TSS
**chr3**	101404551	101406135	TSS
**chr3**	115510829	115511696	TSS
**chr3**	122101419	122102720	TSS
**chr3**	122608308	122609862	Distal
**chr3**	123304120	123305359	TSS
**chr3**	128597133	128598237	TSS
**chr3**	133163026	133163848	Distal
**chr3**	147125039	147126941	TSS
**chr3**	149292776	149294852	Distal
**chr3**	149686486	149687777	TSS
**chr3**	150128819	150133128	TSS
**chr3**	152972520	152976486	Distal
**chr3**	159398625	159400762	Distal
**chr3**	161607089	161608525	Distal
**chr3**	165518483	165520097	Distal
**chr3**	184305610	184309297	Distal
**chr3**	185080246	185082052	TSS
**chr3**	185644039	185647027	TSS
**chr3**	185647213	185648902	TSS
**chr3**	185649771	185655147	TSS
**chr3**	185759558	185764329	Distal
**chr3**	185764536	185767852	Distal
**chr3**	185784225	185789447	Distal
**chr3**	185789997	185794952	Distal
**chr3**	185798535	185800154	Distal
**chr3**	185800551	185803250	Distal
**chr3**	185803827	185820310	TSS
**chr3**	185820471	185826398	TSS
**chr3**	185826983	185829894	TSS
**chr3**	185883335	185884678	Distal
**chr3**	186287514	186288402	TSS
**chr3**	195424399	195428383	TSS
**chr3**	196377219	196378415	Distal
**chr3**	196759936	196760966	Distal
**chr3**	197160644	197161294	Distal
**chr3**	197676176	197678154	TSS
**chr3**	197739160	197742826	Distal
**chr3**	197900029	197900555	Distal
**chr4**	1169498	1171450	Distal
**chr4**	8413479	8414440	Distal
**chr4**	8610294	8611632	Distal
**chr4**	26321702	26322384	TSS
**chr4**	26343972	26344971	Distal
**chr4**	40058970	40060338	TSS
**chr4**	40910947	40912193	Distal
**chr4**	42459505	42461923	TSS
**chr4**	77502150	77507194	TSS
**chr4**	83820649	83821820	TSS
**chr4**	88340552	88342064	Distal
**chr4**	100010038	100010866	TSS
**chr4**	103708500	103709795	Distal
**chr4**	103710018	103714992	Distal
**chr4**	140755655	140756870	Distal
**chr4**	141728638	141729586	Distal
**chr4**	158621752	158624270	Distal
**chr4**	174253332	174254761	TSS
**chr4**	175204493	175206220	TSS
**chr4**	181710769	181712848	Distal
**chr4**	189391497	189392765	Distal
**chr4**	189970158	189972465	TSS
**chr4**	191043604	191044306	Distal
**chr5**	11429	11875	Distal
**chr5**	1456697	1460290	Distal
**chr5**	1541357	1543392	Distal
**chr5**	1543648	1544863	Distal
**chr5**	4369336	4372230	Distal
**chr5**	4547966	4551857	Distal
**chr5**	4575300	4578387	Distal
**chr5**	20972700	20975803	TSS
**chr5**	31935309	31937691	TSS
**chr5**	32026725	32028793	Distal
**chr5**	33439356	33442714	TSS
**chr5**	41056894	41057921	Distal
**chr5**	43066679	43068389	TSS

**chr5**	76085916	76087388	Distal
**chr5**	76507250	76511299	TSS
**chr5**	81692338	81693991	Distal
**chr5**	82321936	82323718	Distal
**chr5**	82801885	82806296	Distal
**chr5**	89705820	89706562	TSS
**chr5**	90676263	90679121	TSS
**chr5**	112439562	112442898	Distal
**chr5**	137513573	137514633	TSS
**chr5**	139510888	139512766	Distal
**chr5**	139935493	139936685	TSS
**chr5**	140900601	140902035	Distal
**chr5**	140937634	140938693	TSS
**chr5**	141704329	141705367	TSS
**chr5**	142076610	142078744	TSS
**chr5**	149685101	149686051	Distal
**chr5**	162930873	162932445	TSS
**chr5**	172741672	172744122	Distal
**chr5**	177632494	177634638	TSS
**chr5**	180670684	180671444	TSS
**chr6**	10395095	10396577	Distal
**chr6**	10405769	10407439	TSS
**chr6**	10408558	10410006	TSS
**chr6**	10527918	10530894	TSS
**chr6**	10555046	10557238	TSS
**chr6**	10612526	10614546	Distal
**chr6**	10641672	10644648	Distal
**chr6**	12013374	12015146	TSS
**chr6**	16344476	16346794	Distal
**chr6**	16412001	16415199	Distal
**chr6**	16423238	16425389	Distal
**chr6**	16758134	16760040	TSS
**chr6**	16801614	16805440	TSS
**chr6**	20533759	20535748	TSS
**chr6**	29562315	29563386	Distal
**chr6**	29563795	29568395	Distal
**chr6**	29592225	29595115	TSS
**chr6**	30582379	30584993	TSS
**chr6**	30686978	30687911	TSS
**chr6**	31508755	31510753	TSS
**chr6**	32937161	32938190	TSS
**chr6**	33238855	33239684	TSS
**chr6**	34855922	34856522	TSS
**chr6**	36562118	36564272	TSS
**chr6**	37211758	37213489	Distal
**chr6**	40407793	40409913	Distal
**chr6**	41977779	41978923	Distal
**chr6**	42417366	42419836	TSS
**chr6**	42660134	42661323	Distal
**chr6**	43594691	43595559	TSS
**chr6**	72674360	72675956	Distal
**chr6**	75952864	75953495	TSS
**chr6**	89791610	89794444	TSS
**chr6**	90007037	90009560	Distal
**chr6**	90926847	90929944	TSS
**chr6**	91073700	91075247	Distal
**chr6**	99872772	99874558	TSS
**chr6**	112651045	112653788	Distal
**chr6**	119493137	119497014	Distal
**chr6**	138737360	138741252	Distal
**chr6**	138780044	138783098	Distal
**chr6**	138817694	138822877	TSS
**chr6**	138835544	138837847	Distal
**chr6**	138842809	138846242	Distal
**chr6**	138874801	138878342	Distal
**chr6**	138880559	138882199	Distal
**chr6**	138891124	138893107	TSS
**chr6**	138913392	138916837	TSS
**chr6**	139011811	139013189	TSS
**chr6**	146610928	146612996	Distal
**chr6**	148841436	148847042	TSS
**chr6**	164497270	164498848	Distal
**chr6**	166289425	166292057	TSS
**chr6**	166395776	166399217	TSS
**chr6**	166399451	166400252	TSS
**chr6**	166419708	166421145	TSS
**chr6**	166878846	166881964	TSS
**chr6**	168904786	168908016	Distal
**chr7**	135845	138157	TSS

**chr7**	8009567	8013264	TSS
**chr7**	13958550	13962532	Distal
**chr7**	14017094	14033096	TSS
**chr7**	23506537	23508121	TSS
**chr7**	23509684	23510659	TSS
**chr7**	23511079	23513111	TSS
**chr7**	30633342	30634401	TSS
**chr7**	30634800	30637145	TSS
**chr7**	31022451	31023345	Distal
**chr7**	35831948	35833655	Distal
**chr7**	38218022	38219806	TSS
**chr7**	42144334	42146423	Distal
**chr7**	42146839	42150659	TSS
**chr7**	51526770	51528749	Distal
**chr7**	75340466	75343015	Distal
**chr7**	76255379	76255989	TSS
**chr7**	90223890	90225088	TSS
**chr7**	100804590	100805698	TSS
**chr7**	100809059	100809770	TSS
**chr7**	100810215	100812133	TSS
**chr7**	104622798	104624647	TSS
**chr7**	106807646	106809443	TSS
**chr7**	107027142	107028643	Distal
**chr7**	114694188	114696691	TSS
**chr7**	114717667	114721514	TSS
**chr7**	128499914	128501155	Distal
**chr7**	129588358	129591123	TSS
**chr7**	138306531	138307063	Distal
**chr7**	138791951	138793209	TSS
**chr7**	154708547	154709529	Distal
**chr7**	157950763	157953053	Distal
**chr8**	1463246	1467015	TSS
**chr8**	1712879	1714434	TSS
**chr8**	6414562	6420047	TSS
**chr8**	22397678	22399027	Distal
**chr8**	22399222	22400303	Distal
**chr8**	24811978	24812778	TSS
**chr8**	25370998	25374669	Distal
**chr8**	25441054	25444176	TSS
**chr8**	25877574	25887096	Distal
**chr8**	27750746	27754148	Distal
**chr8**	27757674	27759687	Distal
**chr8**	27840033	27843391	TSS
**chr8**	29197809	29199644	TSS
**chr8**	29200838	29205391	TSS
**chr8**	29208721	29209529	TSS
**chr8**	29663590	29666229	TSS
**chr8**	29742311	29745319	Distal
**chr8**	48265800	48266988	Distal
**chr8**	61495777	61496984	Distal
**chr8**	61532108	61533252	Distal
**chr8**	61708039	61709288	Distal
**chr8**	61719315	61721841	TSS
**chr8**	66446834	66447906	Distal
**chr8**	67090585	67092290	TSS
**chr8**	116461357	116465154	TSS
**chr8**	121972399	121973380	Distal
**chr8**	125462385	125463380	TSS
**chr8**	125485532	125486815	TSS
**chr8**	128746549	128747781	TSS
**chr8**	129138617	129141148	Distal
**chr8**	130563430	130565232	Distal
**chr8**	130704142	130705842	Distal
**chr8**	130720215	130723479	Distal
**chr8**	135725617	135726950	TSS
**chr8**	140868034	140869676	Distal
**chr8**	140875678	140878097	TSS
**chr8**	141473233	141474869	TSS
**chr8**	141517401	141519124	TSS
**chr9**	14207775	14211760	Distal
**chr9**	14317529	14318445	TSS
**chr9**	27161092	27163086	Distal
**chr9**	33125137	33126934	Distal
**chr9**	35732603	35733467	TSS
**chr9**	73077453	73078555	Distal
**chr9**	78750369	78752388	Distal
**chr9**	86736778	86738194	Distal
**chr9**	91660446	91678111	Distal
**chr9**	91707591	91708721	Distal

**chr9**	91712119	91717990	Distal
**chr9**	91779386	91780685	Distal
**chr9**	91789071	91792472	TSS
**chr9**	91793670	91796451	TSS
**chr9**	91926560	91933279	TSS
**chr9**	98980052	98980460	TSS
**chr9**	127420195	127423664	TSS
**chr9**	127424611	127426894	TSS
**chr9**	128776279	128777113	Distal
**chr9**	140222124	140222934	Distal
**chr9**	140269795	140270629	Distal
**chr9**	140271579	140273767	Distal
**chr10**	1074588	1075500	Distal
**chr10**	1075692	1076977	Distal
**chr10**	3234829	3236532	Distal
**chr10**	4066038	4067634	TSS
**chr10**	4127698	4129401	Distal
**chr10**	4492525	4495503	Distal
**chr10**	6060466	6061728	Distal
**chr10**	13764502	13765884	Distal
**chr10**	18294444	18296030	Distal
**chr10**	21808045	21809335	TSS
**chr10**	21813574	21814839	TSS
**chr10**	22012681	22014502	Distal
**chr10**	65028071	65029558	TSS
**chr10**	75503191	75504529	TSS
**chr10**	79011462	79013411	Distal
**chr10**	79044754	79048401	Distal
**chr10**	79049045	79051008	Distal
**chr10**	82299577	82302812	TSS
**chr10**	98160392	98162279	Distal
**chr10**	99213719	99214507	Distal
**chr10**	99599178	99600786	Distal
**chr10**	102045423	102046122	TSS
**chr10**	104004719	104006281	TSS
**chr10**	104040083	104041062	TSS
**chr10**	111724942	111725868	TSS
**chr10**	111726335	111727882	TSS
**chr10**	115613032	115614558	TSS
**chr11**	1022449	1023723	Distal
**chr11**	1964786	1965815	Distal
**chr11**	2958740	2960430	Distal
**chr11**	2965184	2965669	Distal
**chr11**	2966303	2967864	Distal
**chr11**	3373462	3379170	Distal
**chr11**	7911510	7913776	Distal
**chr11**	22761669	22765518	Distal
**chr11**	27315343	27317945	Distal
**chr11**	32467396	32469312	Distal
**chr11**	33100109	33101478	Distal
**chr11**	33280167	33280885	TSS
**chr11**	33673910	33675132	Distal
**chr11**	34363154	34364328	Distal
**chr11**	35269633	35271280	Distal
**chr11**	36764640	36766678	Distal
**chr11**	43574941	43578107	TSS
**chr11**	43702685	43703986	TSS
**chr11**	46299953	46303851	TSS
**chr11**	46344468	46347764	Distal
**chr11**	46638077	46640787	TSS
**chr11**	47560203	47560833	TSS
**chr11**	47789074	47790174	TSS
**chr11**	57412174	57414442	TSS
**chr11**	57414780	57417233	TSS
**chr11**	57417537	57421313	TSS
**chr11**	58689910	58691260	Distal
**chr11**	62607130	62609477	TSS
**chr11**	65192392	65195480	TSS
**chr11**	65265883	65274193	TSS
**chr11**	67007996	67009044	TSS
**chr11**	69065111	69067043	TSS
**chr11**	70806290	70808332	Distal
**chr11**	74970655	74974120	Distal
**chr11**	76154795	76156284	TSS
**chr11**	77600674	77602776	Distal
**chr11**	94526588	94528523	TSS
**chr11**	95523673	95525403	TSS
**chr11**	115227247	115229099	Distal
**chr11**	117979930	117981028	Distal

**chr11**	118276938	118278177	Distal
**chr11**	122602970	122605382	Distal
**chr11**	122933748	122934948	TSS
**chr11**	125037403	125040762	TSS
**chr11**	125048534	125053004	Distal
**chr11**	125216045	125216666	Distal
**chr11**	125495882	125496739	TSS
**chr11**	126099689	126102552	Distal
**chr11**	128062618	128066209	Distal
**chr11**	128104587	128106819	Distal
**chr11**	128158569	128161746	TSS
**chr11**	128262998	128266455	Distal
**chr11**	128316440	128318211	Distal
**chr11**	128318422	128321307	Distal
**chr11**	128321738	128331863	Distal
**chr11**	128332504	128342770	Distal
**chr11**	128342935	128345452	Distal
**chr11**	128360810	128391610	TSS
**chr11**	128392574	128395929	TSS
**chr11**	128413179	128416452	TSS
**chr11**	128419395	128424760	TSS
**chr12**	95320	95799	Distal
**chr12**	863462	866397	TSS
**chr12**	2364890	2365298	Distal
**chr12**	4223981	4228726	TSS
**chr12**	4242758	4244716	Distal
**chr12**	4244898	4246531	Distal
**chr12**	4384868	4387017	TSS
**chr12**	4396322	4397375	Distal
**chr12**	4401106	4403177	Distal
**chr12**	4413194	4414828	Distal
**chr12**	4415839	4416991	Distal
**chr12**	4647010	4648490	TSS
**chr12**	7052465	7053813	TSS
**chr12**	8057604	8059081	Distal
**chr12**	8090377	8091212	Distal
**chr12**	8233720	8235887	TSS
**chr12**	10873443	10875093	TSS
**chr12**	12657754	12660346	TSS
**chr12**	12867457	12869559	TSS
**chr12**	12871148	12876600	TSS
**chr12**	13317915	13319117	Distal
**chr12**	14795402	14797668	Distal
**chr12**	26081197	26082875	Distal
**chr12**	49462455	49463424	TSS
**chr12**	52279724	52280769	Distal
**chr12**	53873627	53876474	Distal
**chr12**	53876998	53879691	Distal
**chr12**	54672395	54675814	TSS
**chr12**	56390346	56391385	TSS
**chr12**	56509636	56511267	TSS
**chr12**	56552068	56554955	TSS
**chr12**	57106293	57111197	Distal
**chr12**	57117253	57120021	TSS
**chr12**	58138146	58139858	TSS
**chr12**	64798851	64799620	TSS
**chr12**	65720690	65722842	Distal
**chr12**	65903011	65906357	Distal
**chr12**	65996549	65998696	TSS
**chr12**	66220194	66224814	TSS
**chr12**	66225967	66227049	Distal
**chr12**	66228595	66229870	Distal
**chr12**	66234427	66235746	Distal
**chr12**	66239464	66241521	Distal
**chr12**	66270626	66271468	TSS
**chr12**	66271784	66277539	TSS
**chr12**	66284986	66288397	TSS
**chr12**	66292925	66294305	Distal
**chr12**	66323565	66325488	Distal
**chr12**	66326634	66332272	Distal
**chr12**	66333625	66338295	Distal
**chr12**	66338989	66344864	Distal
**chr12**	66347075	66348358	Distal
**chr12**	66349080	66350731	Distal
**chr12**	66354932	66357048	Distal
**chr12**	66358702	66366755	Distal
**chr12**	68099344	68100057	Distal
**chr12**	89743207	89745198	TSS
**chr12**	89745926	89747123	TSS

**chr12**	105802359	105804723	Distal
**chr12**	108997794	109000827	Distal
**chr12**	111298468	111299910	Distal
**chr12**	120729229	120731352	TSS
**chr12**	122239357	122240870	TSS
**chr12**	123010749	123012540	TSS
**chr12**	123942259	123943352	TSS
**chr12**	125399849	125403059	TSS
**chr12**	125411754	125412886	TSS
**chr12**	125422103	125425402	TSS
**chr12**	129192702	129193811	TSS
**chr12**	132172368	132174964	Distal
**chr12**	132478838	132481804	Distal
**chr12**	132873643	132877401	Distal
**chr12**	133188003	133190272	TSS
**chr12**	133190650	133194882	TSS
**chr12**	133219199	133221360	Distal
**chr12**	133221847	133222979	Distal
**chr12**	133249043	133249783	Distal
**chr12**	133255091	133257857	Distal
**chr12**	133258917	133263350	TSS
**chr12**	133265917	133287091	TSS
**chr12**	133288065	133293599	TSS
**chr13**	30130657	30131884	Distal
**chr13**	30607684	30608830	Distal
**chr13**	33924965	33926545	TSS
**chr13**	37573624	37576427	TSS
**chr13**	37632390	37633569	TSS
**chr13**	39771165	39772344	Distal
**chr13**	40110419	40111707	Distal
**chr13**	50017268	50018126	TSS
**chr13**	53225887	53226781	TSS
**chr13**	77902360	77904645	TSS
**chr13**	80932256	80936432	Distal
**chr13**	81009789	81012365	Distal
**chr13**	93961085	93964024	TSS
**chr13**	98796381	98798314	TSS
**chr13**	110352210	110353558	Distal
**chr13**	110428988	110431330	TSS
**chr13**	110699644	110700910	Distal
**chr13**	114553720	114554718	Distal
**chr14**	21561658	21562716	TSS
**chr14**	21670280	21672253	Distal
**chr14**	21735426	21737573	TSS
**chr14**	23789480	23790374	TSS
**chr14**	23987569	23989193	Distal
**chr14**	24701706	24703685	TSS
**chr14**	35449850	35451651	TSS
**chr14**	35589801	35593317	TSS
**chr14**	50412170	50414178	Distal
**chr14**	58763357	58764999	TSS
**chr14**	58893881	58897755	TSS
**chr14**	63670187	63672757	TSS
**chr14**	63761127	63763073	TSS
**chr14**	75229264	75229894	TSS
**chr14**	75232107	75232804	TSS
**chr14**	75746022	75747994	TSS
**chr14**	89793315	89795893	Distal
**chr14**	89805562	89807943	Distal
**chr14**	89894301	89897574	TSS
**chr14**	89908669	89910244	Distal
**chr14**	102276146	102277319	TSS
**chr15**	30917703	30918236	TSS
**chr15**	38545461	38550458	TSS
**chr15**	40737496	40738705	TSS
**chr15**	49201961	49203859	Distal
**chr15**	49255616	49257361	TSS
**chr15**	52943827	52945993	TSS
**chr15**	64993388	64994614	TSS
**chr15**	66661166	66662736	TSS
**chr15**	69743674	69745040	TSS
**chr15**	73988767	73989880	Distal
**chr15**	75015972	75016434	TSS
**chr15**	77779494	77780620	Distal
**chr15**	89740577	89741325	Distal
**chr15**	93424775	93425747	TSS
**chr15**	93443030	93447453	TSS
**chr15**	99341862	99345017	TSS
**chr15**	100257097	100261888	TSS

**chr15**	101441029	101444799	Distal
**chr15**	101727675	101729196	TSS
**chr16**	635537	638551	TSS
**chr16**	895479	897736	Distal
**chr16**	4460141	4461215	Distal
**chr16**	15110053	15111644	Distal
**chr16**	15360671	15362170	Distal
**chr16**	18800046	18801167	TSS
**chr16**	19117117	19118205	Distal
**chr16**	21564388	21565566	Distal
**chr16**	22738365	22740940	Distal
**chr16**	23142817	23144651	Distal
**chr16**	23222376	23223607	Distal
**chr16**	23234897	23237966	Distal
**chr16**	23323359	23325529	Distal
**chr16**	23389801	23391378	Distal
**chr16**	24551646	24553786	TSS
**chr16**	26037594	26039223	Distal
**chr16**	30886822	30887992	TSS
**chr16**	30968193	30969077	TSS
**chr16**	31463104	31464217	Distal
**chr16**	31725050	31725573	TSS
**chr16**	49788297	49790309	TSS
**chr16**	49790627	49791709	TSS
**chr16**	49792704	49793921	Distal
**chr16**	49794264	49796163	Distal
**chr16**	49878036	49879325	Distal
**chr16**	49883354	49888314	TSS
**chr16**	52845956	52848114	Distal
**chr16**	53113166	53115328	TSS
**chr16**	53123790	53126533	TSS
**chr16**	53241887	53243983	TSS
**chr16**	57333334	57334557	TSS
**chr16**	65035404	65036877	Distal
**chr16**	67062141	67062696	TSS
**chr16**	69444047	69444769	Distal
**chr16**	70461810	70463260	TSS
**chr16**	70557676	70558656	TSS
**chr16**	70610267	70611151	Distal
**chr16**	75467522	75468769	TSS
**chr16**	81521228	81524099	Distal
**chr16**	85159119	85159744	Distal
**chr16**	85159898	85161310	Distal
**chr16**	85182205	85183211	Distal
**chr16**	85205912	85208756	TSS
**chr16**	85237186	85240238	TSS
**chr16**	85713370	85715506	Distal
**chr16**	87326864	87329343	TSS
**chr16**	87402765	87403755	TSS
**chr16**	87421487	87422032	TSS
**chr16**	89766313	89766730	TSS
**chr17**	643093	644906	Distal
**chr17**	900896	902520	TSS
**chr17**	903672	904913	Distal
**chr17**	2025917	2027607	TSS
**chr17**	4703800	4704520	Distal
**chr17**	4811564	4812485	TSS
**chr17**	4849553	4850716	TSS
**chr17**	4870350	4871111	TSS
**chr17**	5795997	5801307	Distal
**chr17**	7123732	7124619	TSS
**chr17**	7209983	7210711	TSS
**chr17**	7487174	7487946	TSS
**chr17**	7740005	7741129	TSS
**chr17**	7759758	7760447	TSS
**chr17**	7760628	7761881	TSS
**chr17**	7783857	7787710	TSS
**chr17**	7789701	7790884	TSS
**chr17**	7791201	7793583	TSS
**chr17**	8076491	8077376	TSS
**chr17**	8089736	8091721	TSS
**chr17**	8262226	8264767	TSS
**chr17**	8760037	8763143	Distal
**chr17**	17629234	17630502	TSS
**chr17**	19091257	19091633	TSS
**chr17**	28443529	28444717	TSS
**chr17**	33090194	33092360	Distal
**chr17**	34100599	34102088	TSS
**chr17**	36956393	36957045	TSS

**chr17**	38601077	38601850	TSS
**chr17**	38802985	38804327	TSS
**chr17**	40927365	40928829	Distal
**chr17**	40948735	40950671	TSS
**chr17**	41321911	41323076	TSS
**chr17**	41437470	41439048	TSS
**chr17**	41600547	41605509	Distal
**chr17**	41607377	41614745	TSS
**chr17**	41615121	41617467	TSS
**chr17**	41617698	41622815	TSS
**chr17**	41623739	41625425	TSS
**chr17**	42214807	42216547	Distal
**chr17**	43225182	43225583	TSS
**chr17**	43225820	43226610	TSS
**chr17**	43237322	43238274	TSS
**chr17**	48507272	48510482	TSS
**chr17**	48944513	48946373	TSS
**chr17**	56070838	56072002	Distal
**chr17**	56158365	56159638	TSS
**chr17**	57231684	57232782	TSS
**chr17**	57863824	57864580	TSS
**chr17**	57914486	57918921	TSS
**chr17**	63213571	63215001	Distal
**chr17**	65557653	65559157	Distal
**chr17**	74015285	74016779	Distal
**chr18**	9942	10318	Distal
**chr18**	3453780	3454824	TSS
**chr18**	3593000	3596160	TSS
**chr18**	3602694	3604956	TSS
**chr18**	4643625	4646530	Distal
**chr18**	45084133	45085231	TSS
**chr18**	55447144	55448720	Distal
**chr18**	60524660	60525533	Distal
**chr18**	70322401	70325451	Distal
**chr18**	73946076	73947419	TSS
**chr19**	402241	403747	TSS
**chr19**	1997538	1998719	Distal
**chr19**	2003541	2005501	Distal
**chr19**	2005864	2010272	Distal
**chr19**	2145691	2146544	Distal
**chr19**	4035553	4036975	Distal
**chr19**	4246961	4247311	TSS
**chr19**	8654725	8656731	TSS
**chr19**	8657920	8660456	TSS
**chr19**	8661541	8662692	Distal
**chr19**	8663644	8665294	Distal
**chr19**	8665963	8668057	Distal
**chr19**	8780868	8783474	Distal
**chr19**	8783653	8786665	Distal
**chr19**	8792055	8793640	Distal
**chr19**	9731608	9733400	TSS
**chr19**	9982358	9983937	Distal
**chr19**	10081467	10083390	Distal
**chr19**	10765981	10767477	TSS
**chr19**	12833436	12835091	TSS
**chr19**	13894028	13894557	Distal
**chr19**	13907088	13908213	TSS
**chr19**	15333462	15334243	TSS
**chr19**	15441073	15442222	TSS
**chr19**	38878795	38879246	TSS
**chr19**	38879572	38880932	TSS
**chr19**	38881492	38885198	TSS
**chr19**	38886807	38893575	TSS
**chr19**	39183752	39184705	Distal
**chr19**	41768997	41770261	TSS
**chr19**	41814100	41817146	TSS
**chr19**	41830091	41831043	TSS
**chr19**	41833966	41835133	TSS
**chr19**	42413998	42416664	Distal
**chr19**	45379777	45381237	Distal
**chr19**	45393088	45393901	TSS
**chr19**	45594929	45596393	TSS
**chr19**	45596819	45598213	TSS
**chr19**	49160003	49160826	Distal
**chr19**	50316360	50317326	TSS
**chr19**	52208248	52216385	TSS
**chr19**	52552215	52552870	TSS
**chr19**	58237849	58240496	TSS
**chr20**	1841277	1843372	Distal

**chr20**	1864984	1866210	Distal
**chr20**	2451227	2451884	TSS
**chr20**	4492516	4495848	Distal
**chr20**	17949975	17951048	TSS
**chr20**	30160146	30160770	TSS
**chr20**	30191565	30192908	TSS
**chr20**	35484390	35485774	Distal
**chr20**	39631340	39632529	Distal
**chr20**	39658513	39659906	TSS
**chr20**	46270952	46272703	Distal
**chr20**	47895154	47895814	TSS
**chr20**	52193138	52194310	TSS
**chr20**	52194677	52197961	TSS
**chr20**	52204946	52207025	TSS
**chr20**	52237662	52240973	TSS
**chr20**	52353344	52357737	TSS
**chr20**	52367220	52370353	TSS
**chr20**	52405885	52408341	Distal
**chr20**	52481314	52484603	TSS
**chr20**	52515036	52517763	Distal
**chr20**	52555806	52558582	TSS
**chr20**	59816058	59819962	Distal
**chr20**	59823441	59827026	TSS
**chr20**	59829039	59833275	TSS
**chr20**	59833430	59850207	TSS
**chr20**	59850378	59858007	Distal
**chr20**	59865292	59870704	Distal
**chr20**	59871803	59875255	Distal
**chr20**	59906823	59925047	Distal
**chr20**	59961778	59963300	TSS
**chr20**	59963467	59974143	Distal
**chr20**	59976016	59979184	Distal
**chr20**	59998111	60000195	Distal
**chr20**	60000835	60004638	Distal
**chr20**	60357403	60358807	Distal
**chr20**	60362653	60366676	Distal
**chr20**	60432902	60434706	Distal
**chr20**	60440233	60441741	Distal
**chr20**	60523896	60524531	Distal
**chr20**	60529573	60532754	TSS
**chr20**	61970629	61972138	Distal
**chr21**	27540564	27541817	TSS
**chr21**	33471373	33472752	Distal
**chr21**	33893293	33895015	Distal
**chr21**	34852648	34853409	TSS
**chr21**	34914533	34918283	TSS
**chr21**	43768620	43770104	Distal
**chr21**	44250745	44252288	Distal
**chr21**	44252465	44255099	Distal
**chr21**	44343874	44345054	TSS
**chr21**	44841846	44845774	TSS
**chr21**	45631294	45633383	TSS
**chr22**	18243015	18244174	Distal
**chr22**	19982285	19983207	Distal
**chr22**	22005119	22006461	TSS
**chr22**	25289700	25291620	Distal
**chr22**	25480754	25483096	Distal
**chr22**	38093012	38093683	TSS
**chr22**	39100606	39101774	TSS
**chr22**	43011016	43011982	TSS
**chr22**	45819130	45820618	Distal
**chr22**	46961090	46965063	Distal
**chr22**	49975584	49976363	Distal
**chrX**	41192827	41193777	TSS
**chrX**	54555955	54556470	TSS
**chrX**	116225419	116231040	Distal

Table S5. CIC-DUX4 direct target genes

ACAD9
ACADVL
ACTR8
ADAMTS10
ADH5
AGBL5
AGBL5-AS1
ALG5
ANGPT2
ANKS1A
ANP32E
ARHGAP11B
ARID4A
ARRDC3
ATG13
ATXN7
BLOC1S2
BRD2
BRD8
BTF3L4
C12orf4
C12orf57
CAB39L
CAMK1

CAMK1G
CASC11
CBFB
CBX5
CCDC58
CCDC94
CCDC97
CCNYL1
CDH4
CDK14
CDKAL1
CDKN1B
CDKN2C
CEP44
CEP57
CETN3
CFDP1
CHD2
CHD3
CHEK1
CKS2
CMSS1
CNTD1
COA3
COG4
COX20
COX7A2
CREB3
CREB3L1
CRH
CYB5D1
CYR61
DCLRE1A
DDX39B
DDX39B-AS1
DDX3X
DEDD
DLG4
DLGAP1-AS1
DLGAP1-AS2
DNAJB11
DNAJB4
DUSP4
DUSP6
EIF2AK3
EIF5A
EPC2
ERVH48-1
ETS1
ETV1
ETV4
ETV5
EXOSC8
FAM162A
FAM214A
FAM228B
FAM76B
FAM98C
FBXO8
FGF1
FNBP4
FOS
FUBP1
FUZ
GABBR1
GABPB2
GARS
GART
GATAD2B
GBF1
GCNT2
GGN
GMPR2
GNL3L
GPATCH4
GPN1
GTPBP1
GTPBP2
HARBI1
HBP1
HEXIM1
HEXIM2
HIPK3
HIVEP1
HM13-AS1
HMGB2
HNRNPA1
HNRNPA1P10
HNRNPAB
HNRNPC
HNRNPUL1
HSD17B12
HSPA8
ID1
IGF2BP3
IQCG
JMJD1C
KDM2A
KIAA0391
KIAA0586
KIF20A
KLF7
KNTC1
LEPR
LRCOL1
MAGOH
MALAT1
MAP3K13
MAST2
MAT2B
MGME1
MIR1257
MIR181A2HG
MIR21
MIR301B
MIR3153
MIR3175
MIR3184
MIR423
MIR4279
MIR4519
MIR5188
MIR570
MPDU1
MPV17
MRPS18B
MYC
MYL6
MYLK-AS1
N4BP2
NAA38
NACA
NBR1
NECAP1
NEDD8
NEDD8-MDP1
NHLRC2
NHSL1
NIF3L1
NOP58
NSRP1
NUCKS1
OAZ2
P2RX2
PABPN1
PCNP
PDE12
PDE8B
PEX19
PFN2
PGAM5
PGAP1
PIP4K2B
PNISR
POLDIP3
POLE
POMZP3
PPIL3
PPP1R10
PPP1R37
PPP2R3C
PPP2R5C
PRR11
PXDN
PXMP2
RAB40C
RAD51AP1
RBBP6
RBPJ
RHEBL1
RHOJ
RNF139
RNF139-AS1
RNU12
RPL24
RPL35A
RPL41
RPLP1
RPRD2
RPS15A
RPS18
RPS6KA2-IT1
RPSAP52
RSRC2
SEC24C
SEC31A
SECISBP2
SETD1A
SETD5
SETDB2
SF3B3
SH2D4B
SHC3
SHC4
SKA2
SKIDA1
SMARCE1
SNORD12C
SNORD84
SNORD95
SNRNP35
SNRPB
SNX5
SON
SPAG7
SPRED1
SPRED3
SPRY4
SRA1
SRP54
SRSF3
STARD3NL
SUGT1
SUOX
SUPT20H
TAF11
TARS
TBCCD1
TGIF1
THAP9
THAP9-AS1
TIMM22
TIMM9
TLN1
TMEM50B
TNPO2
TOB1
TOB1-AS1
TOMM40
TRA2B
TRERF1
TRIOBP
TRMT12
TSPAN31
TTC32
TUBB
TXNDC12

UBC
USF1
VGF
VPS52
WDR74
XPOT
YBX1
YBX3
YLPM1
YPEL4
ZC3H10
ZFAS1
ZFAT
ZIC1
ZIC4
ZNF432
ZNF561
ZNF671
ZNF720
ZNFX1
ZSWIM4

Table S6. Clinicopathological information of primary CIC-DUX4 frozen samples

Sample name	Age at diagnosis	Gender	Tumor location	Tumor size	Metastasis
CIC1	29	F	thigh	3.7 cm	No
CIC2	66	M	neck	6.1 cm	No
CIC3	25	F	thigh	7.5 cm	lungs
